# Impacts of active school design on school-time sedentary behavior and physical activity: A pilot natural experiment

**DOI:** 10.1371/journal.pone.0189236

**Published:** 2017-12-07

**Authors:** Jeri Brittin, Leah Frerichs, John R. Sirard, Nancy M. Wells, Beth M. Myers, Jeanette Garcia, Dina Sorensen, Matthew J. Trowbridge, Terry Huang

**Affiliations:** 1 HDR, Department of Built Environment Research, Omaha, NE, United States of America; 2 University of Nebraska-Lincoln, College of Architecture, Department of Interior Design, Lincoln, NE, United States of America; 3 University of North Carolina-Chapel Hill, School of Public Health, Department of Health Policy and Management, Chapel Hill, NC, United States of America; 4 University of Massachusetts-Amherst, School of Public Health and Health Sciences, Department of Kinesiology, Amherst, MA, United States of America; 5 Cornell University, College of Human Ecology, Department of Design and Environmental Analysis, Ithaca, NY, United States of America; 6 University of Central Florida, College of Education and Human Sciences, Department of Educational and Human Services, Orlando, FL, United States of America; 7 VMDO Architects, Charlottesville, VA, United States of America; 8 University of Virginia, School of Medicine, Department of Emergency Medicine, Charlottesville, VA, United States of America; 9 City University of New York, School of Public Health, Graduate Center, New York, NY, United States of America; University of Auckland, NEW ZEALAND

## Abstract

**Background:**

Children spend a significant portion of their days in sedentary behavior (SB) and on average fail to engage in adequate physical activity (PA). The school built environment may influence SB and PA, but research is limited. This natural experiment evaluated whether an elementary school designed to promote movement impacted students’ school-time SB and PA.

**Methods:**

Accelerometers measured SB and PA at pre and post time-points in an intervention group who moved to the new school (n = 21) and in a comparison group experiencing no school environmental change (n = 20). Difference-in-difference (DD) analysis examined SB and PA outcomes in these groups. Measures were also collected post-intervention from an independent, grade-matched group of students in the new school (n = 21).

**Results:**

As expected, maturational increases in SB were observed. However, DD analysis estimated that the intervention attenuated increase in SB by 81.2 ± 11.4 minutes/day (*p*<0.001), controlling for time in moderate to vigorous physical activity (MVPA). The intervention was also estimated to increase daily number of breaks from SB by 23.4 ± 2.6 (p < .001) and to increase light physical activity (LPA) by 67.7 ± 10.7 minutes/day (*p*<0.001). However, the intervention decreased MVPA by 10.3 ± 2.3 minutes/day (*p*<0.001). Results of grade-matched independent samples analysis were similar, with students in the new vs. old school spending 90.5 ± 16.1 fewer minutes/day in SB, taking 21.1 ± 2.7 more breaks from SB (*p*<0.001), and spending 64.5 ± 14.8 more minutes in LPA (*p*<0.001), controlling for time in MVPA. Students in the new school spent 13.1 ± 2.7 fewer minutes in MVPA (*p*<0.001) than their counterparts in the old school.

**Conclusions:**

This pilot study found that active school design had beneficial effects on SB and LPA, but not on MVPA. Mixed results point to a need for active classroom design strategies to mitigate SB, and quick access from classrooms to areas permissive of high-intensity activities to promote MVPA. Integrating active design with programs/policies to promote PA may yield greatest impact on PA of all intensities.

## Introduction

Physical activity (PA) has profound impacts on children’s current and future health [1], and is positively associated with classroom behavior and learning [2,3]. PA decreases over time in children both prior to and during adolescence [4–11]. It has been shown that children can be sedentary for up to 70% of school time, including physical education class and breaks [12]. Children spend a large proportion of their waking hours in school, and schools are relatively accessible venues for population-based interventions [13]. Therefore, increasing children’s PA at school has become a national focus to address childhood obesity and related diseases. The National Academy of Medicine has emphasized a need to develop high-quality research on the influence of school environments on children’s PA [1].

A number of studies of school built environment characteristics and children’s PA have measured MVPA, with a few also including SB as an outcome [14]. One systematic review concluded that provision of activity-oriented facilities, such as gymnasiums and sports fields, was positively associated with PA during recess [15]. Among longitudinal studies addressing school built environment effects on PA, environmental variables have included exterior features such as gardens [16], playgrounds and outdoor recreation and recess areas [17,18], and the conduciveness of school surroundings for active commuting [18]. A recent playground reconstruction study found that the environmental intervention did not affect MVPA, but increased light PA (LPA) and decreased SB in younger children [19]. Several small-scale longitudinal studies have addressed PA and school classroom features intended to reduce students’ time spent sitting. A review of 13 studies concluded that a range of classroom design approaches, including incorporation of stand-biased and ergonomic versus conventional furnishings, were effective in reducing students’ daily sitting time by 44–60 minutes [20].

This pilot natural experiment, including pre- and post- intervention measures and measures in a grade-matched comparison group, contributes new findings about the impacts of an elementary school environment that was holistically designed to mitigate SB and promote PA. The aim of this study was to test hypotheses that children exposed to a new activity-promoting school built environment would demonstrate, relative to a comparison group, (1) decreased daily school time in SB and more frequent breaks from SB (i.e., transitions to higher intensity activity) as compared to SB time and breaks in their previous traditional school environment, and (2) increased daily school time in LPA and MVPA compared to time spent in these activity intensity levels in their previous school. We also hypothesized that an independent, grade-matched sample of students in the new school would demonstrate lower daily SB time and breaks, and more time in LPA and MVPA, compared to intervention students at baseline in the old school.

## Methods

### Research design

The quasi-experimental research design included pre and post measurements in an intervention group at Virginia elementary schools, and in a comparison group at New York state elementary schools. The comparison group was selected based upon rural location and population demographics similar to those of the intervention group, as well as age of school facility. The comparison group school facilities were conventional decades-old buildings, similar to the baseline school in the intervention group. The comparison group for this study was part of a control group in a separate study of impacts of school garden installation on PA [16].

**Fig 1:**
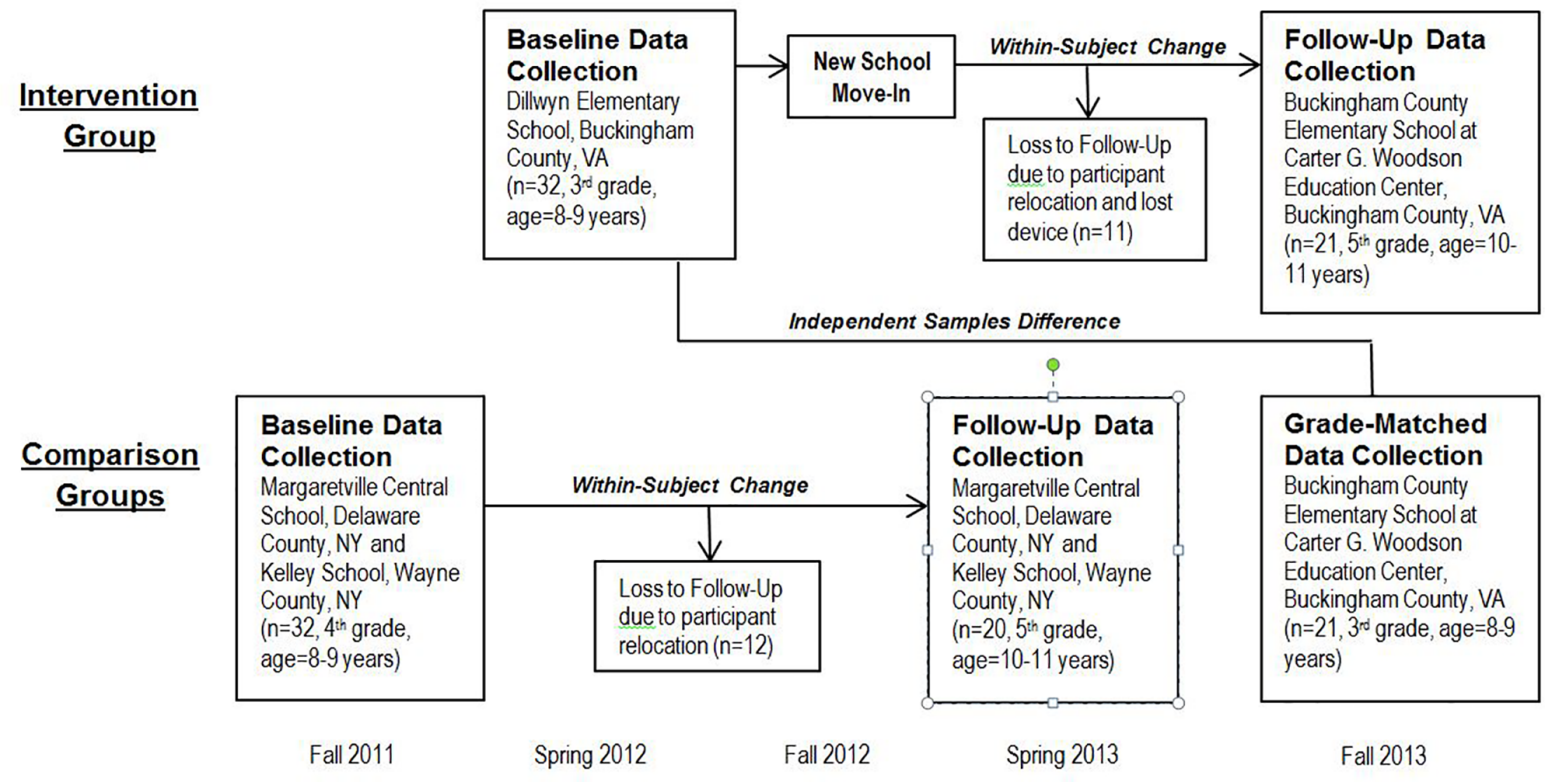
Research design and flowchart of participants.

In addition, an independent sample of students in the new Virginia elementary school, grade-matched to intervention group students measured at baseline, was included at post-intervention to assess the role of other potential confounding effects in outcome changes over time (Fig 1).

### Setting and participants

The intervention and comparison populations both resided in Eastern U.S. rural areas with weather conditions allowing outdoor activities during the periods of data collection. At Buckingham County Elementary School, near the town of Dillwyn, VA, 74% of students in the total student population were eligible for the free and reduced price meal (FRPM) program. In the village of Newark, Wayne County, NY, 56% of students were FRPM-eligible at the Kelley Elementary School. In the village of Margaretville, Delaware County, New York, 55% of students were FRPM-eligible at the Margaretville Central School. Both intervention and comparison schools included notable proportions of minority students (45%, 23%, and 26% of the total school populations above, respectively).

In May 2012, Virginia intervention group data were collected from one arbitrarily selected 3^rd^ grade classroom at Dillwyn Elementary School, which was subsequently closed at the end of the school year. These children moved to the newly designed Buckingham County Elementary School in September 2012. In October 2013, approximately 14 months post-occupancy, data were collected from the same children in three 5^th^ grade Buckingham County Elementary School classrooms. Comparison group data were collected in Wayne and Delaware counties in New York State, first in October and November 2011 in 4^th^ grade classrooms, and again from the same children in May 2013 in 5^th^ grade classrooms at the same schools. These schools did not undergo any renovations during the study time period. Data were also collected in October 2013 from an independent sample of Virginia 3^rd^ graders, in an arbitrarily selected classroom in the new school (Fig 1).

### Environmental intervention

The Virginia baseline data collection occurred at the aging Dillwyn Elementary School, which lacked a gymnasium or other indoor PA-dedicated areas. The facility, a single story structure of 25,246 gross square feet (GSF), was insufficiently sized to accommodate the student population, and trailers supplemented classroom space. The new Carter G. Woodson Education Complex, containing the Buckingham County Elementary School, a two-story structure of 134,015 GSF, was more than 5 times larger and encompassed complete renovation of two previously vacant facilities as well as new construction. Given the shared interest of the architects and school leadership in the opportunity to create a health-promoting school, the architects engaged with public health researchers to design and implement an evaluation in conjunction with the architectural project. We have discussed the challenges and benefits of this inter-disciplinary collaboration elsewhere [14,21]. Design decisions for the new facility drew upon the *Physical Activity Design Guidelines for School Architecture* [14]. Outdoor classrooms, gardens, nature trails, and other landscape amenities were designed to provide active learning opportunities. The facility also included gymnasia, playgrounds, and two large sports fields. The central locations of shared areas such as the Dining Commons and Music and Art Studios were intended to promote walking during the school day. In order to support desirable SB accumulation patterns, classrooms were amply sized to include activity areas and to afford space for movement during class time, with an average of 810 square feet per classroom, or nearly 34 square feet per student. Classroom spaces were outfitted with mobile and dynamic furniture, including height-adjustable chairs that tip, rock, and accommodate forward- or backward-facing sitting positions, sit-to-stand mobile and surface angle adjustable tables for all students. In addition, a mobile screen, stools (footrest and seating, including “wiggle” stools with rounded bottoms), soft seating, and beanbags were made available in each classroom. Specification of dynamic furniture was intended to facilitate children’s natural inclinations to fidget, and to discourage bouts of static sitting. Mobile furnishing afforded many options in classroom layout and group configurations. Further details and illustrations of the intervention school design have previously been published [14].

### Accelerometry procedures and data processing

During each data collection time period, both Virginia and New York school children wore either the ActiGraph GT3X+ or GT1M accelerometer on a belt around the waist, positioned at the right hip bone. Due to high measurement agreement between these two accelerometer models in children, it is acceptable to use both models in a single study [22]. Accelerometry data processing conformed to accepted standards [23], and used ActiLife v.6.11.7 software (ActiGraph Corporation, Pensacola, FL). At each data collection time point, Virginia children wore accelerometers for 5–7 consecutive days including school and home time, and New York children for 3 consecutive days during school time. In this study, all data were filtered for school days and times only, and the valid day definition was set to this pre-set length of the school day for each location. Thus school wear time differed by school, but was consistent across all students within a school. A valid day was defined specifically as the total daily possible wear time of 420 minutes in Buckingham County, VA, 300 minutes in Delaware County, NY, and 360 minutes in Wayne County, NY. Non-wear time definition was 30 consecutive minutes of zero activity counts, and minimum number of valid wear days was three. No non-wear time was identified in the data sets. The wear time variable was included in statistical models, but was non-significant. Evenson et al. (2008) cut points defined SB, and light, moderate, and vigorous activity categories as 0–100 counts per minute (CPM), 101–2295 CPM, 2296–4011 CPM, and 4012+ CPM respectively [24,25]. Minimum length of a sedentary bout was 1 minute. Sixty-second epochs were used in processing the accelerometer data.

### Measures

Mean outcome measures from ActiLife-scored accelerometry data were as follows: number of daily sedentary bouts, average length of sedentary bout (natural log-transformed), number of daily breaks from SB, minutes of SB per day, minutes of LPA per day, and minutes of MVPA per school day. We also performed calculations of metabolic equivalent of task minutes (MET-mins) using the midpoint of 2.25 METS (range of 1.5 to 3.0) for LPA, and the midpoint of 6.0 METS (range of 3.0 to 9.0) for MVPA.

### Statistical analysis

Adequate distributional normality of variables or their natural log-transformed values were confirmed with absolute values of skewness and kurtosis <1. Initial paired-sample t-tests, and then linear mixed models controlling for gender and a binary race/ethnicity variable (minority or white/non-Hispanic), were run to assess within-subject changes in outcomes over time for the intervention and comparison groups separately. Then, difference-in-difference (DD) analyses were conducted to examine net effect of the environmental intervention on SB and PA. The DD method is commonly used in natural experiments to compare change in the outcome in the intervention versus comparison group, under the assumption that the differences between groups would have remained constant under no treatment [26]. Thus, linear mixed models were used to estimate the effect of the intervention by examining the interaction term of time (baseline, follow-up) and group (intervention, comparison), controlling for gender, race/ethnicity, and wear time. In addition, for other outcomes, time in MVPA was included in models to estimate effect sizes that were independent of MVPA.

To assess other potential confounding effects, linear models were used to estimate differences in outcomes between the independent samples of grade-matched students in the old/baseline and new/intervention Virginia schools, controlling for gender, race/ethnicity, and time in MVPA.

Statistical analyses were conducted with SAS v.9.4 software (SAS Institute, Cary, NC).

### Human subjects review

The Institutional Review Boards (IRB) of the University of Virginia and the University of Nebraska Medical Center approved the research protocol for the Virginia students. Parents provided signed informed consent, and students provided verbal assent for participation. For the New York State student samples, the Cornell University IRB deemed the school-time-only protocol exempt, and did not require parental consent or student assent [16].

## Results

There was similar loss to follow-up in the intervention and comparison groups (baseline/follow-up intervention group N = 32/21; comparison group N = 32/20), due primarily to students’ moves to other communities. The Virginia intervention group included a higher proportion of males (70%) versus females, while the New York state comparison group was more gender-balanced (45% male). Age ranges were similar between the two groups, with a one semester offset in data collection timing and same follow-up interval timing. Samples were 52% minority in Virginia, and 10% minority in New York, due to classroom proportions varying from those of the school populations. The independent sample of Virginia 3^rd^ graders in the new school (n = 21) was balanced by gender (48% male), and had 20% minority representation (Table 1).

**Table 1:** Sample demographics.

Data Collection Groups and Timing	N	Age Integer Years (%)	Gender		Race/Ethnicity^a^	
			Female n (%)	Male n (%)	White, Non-Hispanic n (%)	Minority n (%)	
**Comparison Group–Baseline (1**^**st**^ **Semester 4**^**th**^ **Graders)**						
Delaware County, NY (Nov 8–10, 2011) and Wayne County, NY (Oct 4–6, 2011)	32	8 (9%) 9 (91%)	17 (53.1%)	15 (46.9%)	25 (78.1%)	6 (18.8%)	
**Intervention Group–Baseline (2**^**nd**^ **Semester 3**^**rd**^ **Graders)**						
Buckingham County, VA (May 28-Jun 1, 2012)	32	8 (15%) 9 (85%)	10 (31.3%)	22 (68.7%)	13 (40.6%)	16 (50.0%)	
**Comparison Group–Follow-Up (2**^**nd**^ **Semester 5**^**th**^ **Graders)**						
Delaware County, NY (May 13–15, 2013) and Wayne County, NY (May 28–30, 2013)	20	10 (30%) 11 (70%)	11 (5.50%)	9 (4.50%)	16 (80.0%)	4 (20.0%)	
**Intervention Group–Follow-Up (1**^**st**^ **Semester 5**^**th**^ **Graders)**						
Buckingham County, VA (Oct 17–23, 2013)	21	10 (81%) 11 (19%)	6 (28.6%)	15 (71.4%)	10 (47.6%)	11 (52.4%)	
**Grade-Matched Group–Follow-Up (1**^**st**^ **Semester 3**^**rd**^ **Graders)**						
Buckingham County, VA (Oct 17–23, 2013)	21	8 (100%)	11 (52.4%)	10 (47.6%)	13 (61.9%)	4 (19.0%)	

^a^ Black/African-American, Hispanic/Latino, and Other/Mixed Race were combined into one Minority category for analysis. The Intervention group was 47.6% Black/African-American and 4.8% Hispanic/Latino. The Comparison group was 10.0% Black/African-American and 10.0% Hispanic/Latino. Some race/ethnicity data was not reported.

### School-time sedentary behavior

**Fig 2:**
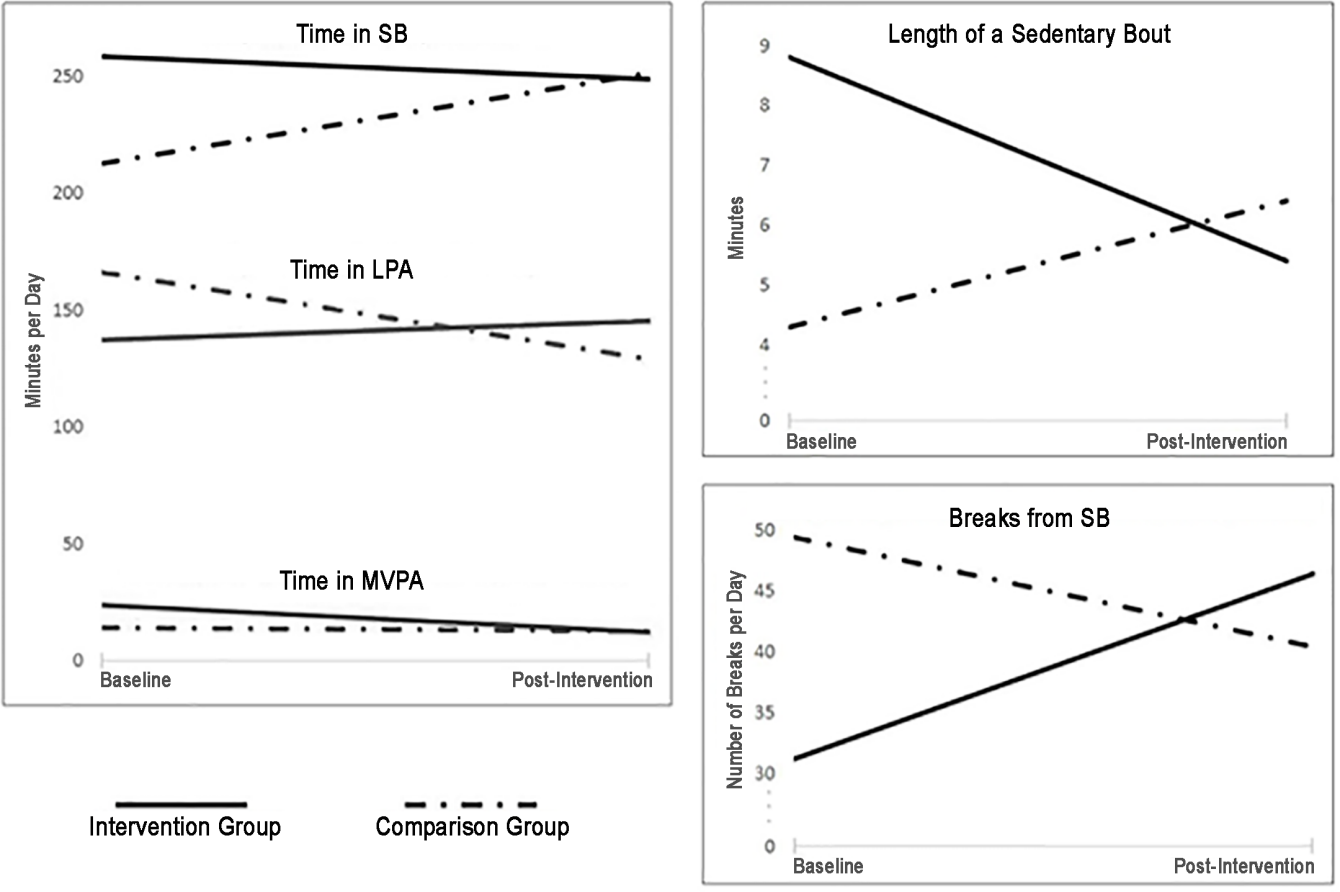
Baseline and post-intervention outcomes of intervention and comparison groups.

In all groups at both baseline and follow-up, mean sedentary time represented more than half (50.7%-63.1%) of the school day. At baseline, the Virginia intervention group had higher time in SB than the New York state comparison group. Post-intervention, the intervention group had similar or lower SB measures versus the comparison group (Fig 2). Separate linear mixed models for each group indicated that change in SB time in the intervention group was non-significant (-13.3 ± 9.0 minutes/day, *p* = 0.154), and that SB time increased in the comparison group (46.7 ± 8.4 minutes/day, *p*<0.001) as expected with age.

Accounting for the baseline between-group difference, DD analyses estimated the intervention’s net effects to decrease SB time by 81.2 ± 11.4 minutes/day (*p*<0.001), to decrease average length of a sedentary bout (estimate based on log-transformed variable, *p*<0.001), and to increase daily number of breaks in SB by 23.4 ± 2.6 (*p*<0.001) (Table 2), all controlling for wear time and time in MVPA. Subsequent analyses including interaction terms of MVPA with group (intervention, comparison) and time (baseline, follow-up) found that change in MVPA did not explain changes in these sedentary pattern variables.

**Table 2:** Difference-in-difference (DD) estimates of the impact of school architecture intervention effects on sedentary behavior and physical activity.

Outcome Variable	Model Controlling for Gender, Race/Ethnicity^a^, and Time in MVPA^b^		
	Parameter Estimate	SE	*p*-Value
**Daily Minutes in Sedentary Behavior**			
Group (Ref: Comparison)	142.4	15.1	<0.001
Time (Ref: Baseline)	27.7	7.4	<0.001
Group*Time (DD effect)	-81.2	11.4	<0.001
**Average Length of a Sedentary Bout**			
Group (Ref: Comparison)	1.02^c^	0.15^c^	<0.001
Time (Ref: Baseline)	0.35^c^	0.07^c^	<0.001
Group *Time (DD effect)	-1.08^c^	0.11^c^	<0.001
**Average Daily Number of Breaks from Sedentary Behavior**			
Group (Ref: Comparison)	-9.5	3.5	0.008
Time (Ref: Baseline)	-6.2	1.7	0.006
Group *Time (DD effect)	23.4	2.6	<0.001
**Average Daily Minutes in LPA**			
Group (Ref: Comparison)	-39.2	14.5	0.009
Time (Ref: Baseline)	-27.6	7.0	0.002
Group *Time (DD effect)	67.7	10.7	<0.001
**Average Daily Minutes in MVPA**			
Group (Ref: Comparison)	10.9	3.6	0.004
Time (Ref: Baseline)	-2.3	1.6	0.224
Group *Time (DD effect)	-10.3	2.3	<0.001

^a^ Race/ethnicity a dichotomous variable with values White/Non-Hispanic or Minority.

^b^ Estimates and *p*-values from linear mixed models of outcomes with time (baseline, follow-up), controlling for differences in accelerometer in-school wear time, gender, race/ethnicity, and time in MVPA for other outcomes.

^c^ Based on natural log transformed variable values.

Third graders in the new Virginia school spent, on average, 90.5 ± 16.1 minutes less daily time in SB than their same-grade counterparts in the old school environment (*p*<0.001), and had shorter sedentary bouts (estimate based on log-transformed variable, *p*<0.001) and 21.1 ± 2.7 more daily breaks in SB (*p*<0.001) (Table 3), controlling for time in MVPA.

**Table 3:** Grade-matched independent samples differences in sedentary behavior and physical activity.

Outcome Variable and Groups			Model Controlling for Gender, Race/Ethnicity^a^, and MVPA^b^		
	Old School Mean (SD) (n = 32)	New School Mean (SD) (n = 21)	Parameter Est. (New vs. Old School)	SE	*p*-Value
**Average Daily Minutes in Sedentary Behavior**					
Grade-Matched Groups	265.2 (39.7)	214.9 (37.6)	-90.5	16.1	<0.001
**Average Length of a Sedentary Bout**					
Grade-Matched Groups	9.2 (4.2)	4.4 (1.0)	-0.95^c^	0.13^c^	<0.001
**Average Daily Number of Breaks from Sedentary Behavior**					
Grade-Matched Groups	30.4 (6.6)	49.0 (18.6)	21.1	2.7	<0.001
**Average Daily Minutes in LPA**					
Grade-Matched Groups	129.8 (34.2)	167.2 (35.3)	64.5	14.8	<0.001
**Average Daily Minutes in MVPA**					
Grade-Matched Groups	25.0 (9.6)	11.2 (4.9)	-13.0	2.7	<0.001

^a^ Race/ethnicity a dichotomous variable with values White/Non-Hispanic or Minority.

^b^ Estimates and *p*-values from linear models of outcomes with group, gender, race/ethnicity, and time in MVPA for other outcomes.

^c^ Based on natural log transformed variable values.

### School-time physical activity

At baseline, the intervention group spent less daily time in LPA and more time in MVPA than the comparison group. Post-intervention, the intervention group spent more daily time in LPA, and similar time in MVPA versus the comparison group (Fig 2). Separate linear mixed models estimated change in LPA time as non-significant in the intervention group (14.1 ± 9.2 minutes/day, *p* = 0.138), and showed a decrease in comparison group LPA time (-36.5 ± 7.9 minutes/day, *p*<0.001).

Accounting for between-group baseline differences, DD analyses estimated net effects of the intervention to increase time in LPA by 67.7 ± 10.7 minutes/day (*p*<0.001), controlling for daily wear time and time in MVPA, and to decrease time in MVPA by 10.3 ± 2.3 minutes/day (*p*<0.001) (Table 2), controlling for daily wear time. This additional 67.7 minutes of LPA equated to 152.3 metabolic equivalent of task minutes (MET-min), more than offsetting the reduction in MVPA of 10.3 minutes or 61.8 MET-min.

The 3^rd^-grade independent sample in the new school spent on average 64.5 ± 14.8 more daily minutes in LPA (*p*<0.001), controlling for time in MVPA, and 13.0 ± 2.7 fewer daily minutes in MVPA, compared to their same-grade counterparts in the old school (*p*<0.001) (Table 3).

## Discussion

This pilot natural experiment with a holistic movement-oriented school built environmental intervention used both longitudinal within-subject and grade-matched independent samples analyses to evaluate intervention effects on students’ SB and PA during school time. The study contributes to a limited body of evidence about the impact of the school built environment on students’ SB and PA, with a research design that facilitates a degree of reasonable causal inference. Results confirmed previous research findings that, on average, children spend a majority of the school day sedentary. DD analyses revealed that the intervention prevented expected maturational SB increases and LPA decreases, and same-grade samples analysis showed improved SB accumulation patterns and higher LPA time in the new school environment. Contrary to expectations, however, the intervention decreased daily time in MVPA, although overall MET-mins increased. Analyses also showed that changes in time in MVPA did not explain changes in SB patterns.

Much of the evidence pertaining to children’s PA, weight status and cardio-metabolic health has focused on moderate-to-vigorous physical activity (MVPA), given its well-documented associations with health indicators, and the U.S. recommendation that children spend at least 60 minutes per day in MVPA [27–29]. Numerous studies have also addressed impacts of sedentary behavior (SB) on children’s health, but its influence, independent of MVPA, has not been fully resolved. While one systematic review concluded that reductions in SB correlated to lower health risk in 5–17 year-old youth [30], a meta-analysis of pooled youth accelerometry data concluded that MVPA was associated with better cardio-metabolic risk factors regardless of time in SB [31]. Other subsequent reviews called for more quality evidence to document MVPA-independent associations of patterns and volumes of child and adolescent SB with health indicators [32], and concluded that evidence for a causal relationship between SB and biomedical and mental health was generally unconvincing due to inconsistent or null findings [33,34]. Therefore, the clinical relevance of changes in SB found in this study remains unclear.

Classrooms, where students typically spend the most time, can be considered as one level of environmental intervention. The intervention effects of reducing time in SB and increasing frequency of transition between SB and higher intensity activity could be related to classroom design that provided dynamic furniture, ample space for activity areas and moving around the classroom, and potential to stand while working, supporting classroom-based findings from previous studies [20]. Anecdotally, the researchers were told that many intervention school teachers designed interactive curricula and activities made possible by the new classroom design and flexible and mobile furnishings, which may have worked in synergy with built environment features to improve SB outcomes.

Analyses showed an increase in LPA and a decrease in MVPA in the intervention school. There were no between-facility differences in school policies or practices (such as time devoted to recess or physical education) that would help explain these outcomes, and researchers on site did not observe particular weather or other circumstances precluding usual daily routines. Results may have been influenced by school-level changes, and specifically the larger size of the new school building and campus. Research has documented positive school and campus size and PA associations in slightly older students [35]. However, many teachers in the intervention school expressed dissatisfaction with long walking distances and time required to reach daily destinations such as recess areas. At a pace required to keep young children together, such walking could have contributed to time in LPA. However, time spent walking may have unintentionally cut into scheduled times for higher-intensity activities. Smaller, more dispersed, high-intensity activity areas may be a worthwhile school design opportunity.

It has been established that children across age groups are more physically active outdoors versus indoors [36–38]. Therefore, school design should consider quick outdoor access to promote moderate-to-vigorous intensity activities that are prohibited in most areas of the school interior. Given baseline MVPA measures, it is possible that the Virginia sample was unusually active compared to the school population mean at that time. The baseline classroom was in a temporary trailer on the school site that provided very quick access to outdoor and recess areas. In the new school, the classroom-to-playground route was more than twice as long and mostly inside. Given a school policy of ‘speeding tickets’ for running in the building, these differences in distances could have impacted both LPA and MVPA.

This study has several limitations that should be considered. Although sample sizes were small, they did provide adequate statistical power to detect highly significant within-subject changes and between-group differences. Results may not necessarily generalize to dissimilar populations, and cross-study comparisons should take into account this study’s methodological choices, such as school-time only outcome measures and 60-second epoch length. These data and analyses provided no inferences about potential impacts on SB or PA outside of school time, and it is possible that MVPA was underestimated due to intensity averaging across 60-second epochs [39]. Although this epoch length has been common in the literature, there has been a recent trend toward use of shorter epoch lengths in children to better capture short spikes of high-intensity activity [23,40]. In addition, body mass index (BMI) data were not made available, and therefore were not included in statistical models. Actual time spent in recess and PE was also not measured, and specific details of adherence to these and other programmatic interventions may be important to include in future studies. As the intervention was a holistic school built environmental change, it was not possible to analyze individual effects of particular environmental variables quantitatively. A further limitation of the study is that, without randomization, regression to the mean cannot be entirely ruled out as a potential concern. However, several outcomes clearly and consistently changed from less to more favorable in the intervention vs. comparison group, mitigating concern about trend toward the mean. In addition, results were consistent in the grade-matched independent samples comparison. Interventions in the built environment are difficult to randomize, and a strength of this study was the use of a similar comparison group, with additional same-grade independent samples analyses to identify potential threats to internal validity.

## Conclusion

Considering the strengths and limitations of this pilot natural experiment, results provide preliminary evidence that active school design can play a role in influencing children’s SB and PA. We documented significant changes in students’ SB patterns and PA after a move to a new school environment designed explicitly to mitigate SB and promote PA. Active classroom design strategies may have had positive impact on school-time SB and LPA accumulation. The large school footprint and long distances to high-intensity activity destinations could have negatively impacted MVPA accumulation. Future longitudinal studies may well engage larger, more representative samples, and employ school/cluster randomization to produce stronger causal evidence. Such studies may also evaluate impacts of coordinated built environment and programmatic interventions intended to maximize use of active school environmental affordances in child and youth populations.

## Supporting information

S1 File SAS dataset contents. (DOCX)

S2 File SAS dataset. (SAS7BDAT)
