# Supplementary material for: Impacts of active school design on school-time sedentary behavior and physical activity: A pilot natural experiment
Source: PLoS One. 2017 Dec 7;12(12):e0189236. doi: 10.1371/journal.pone.0189236 (PMC5720751; doi:10.1371/journal.pone.0189236)
Supplement: S1 File — (DOCX) [file pone.0189236.s001.docx]

| **SAS Dataset VANYACCELDATA Alphabetic List of Variables and Attributes** | | | | |
| --- | --- | --- | --- | --- |
| **#** | **Variable** | **Type** | **Len** | **Label** |
| **6** | age | Num | 8 | Age in Whole Years |
| **7** | ave_mins_sedbout | Num | 8 | Average Length (Minutes) of Sedentary Bouts |
| **12** | avnum_dly_sedbrks | Num | 8 | Average Number of Daily Breaks from SB |
| **11** | daymins | Num | 8 | Accerometer Wear Time (minutes) per Day |
| **5** | ethnicbin | Num | 8 | Binary Race/Ethnicity 1=White/Non-Hispanic 2=Minority (includes Black/African American, Hispanic/Latino, Asian, Other or Mixed Race) |
| **2** | gender | Num | 8 | Gender 1=Female 0=Male |
| **8** | grade | Num | 8 | Grade in School |
| **9** | log_ave_mins_sedbout | Num | 8 | Natural Log Transformation of Average Length of Sedentary Bouts |
| **14** | mins_LPA_day | Num | 8 | Average Total Minutes per Day in LPA |
| **13** | mins_MVPA_day | Num | 8 | Average Total Minutes per Day in MVPA |
| **15** | mins_SB_day | Num | 8 | Average Total Minutes per Day in SB |
| **1** | nid | Num | 8 | Individual ID |
| **4** | school | Num | 8 | School Location 1=Virginia/Buckingham County 2=NewYork/Wayne County/Kelly 3=New York/Delaware County/Margaretville |
| **3** | state | Num | 8 | Group by State & Intervention Status 1=Virginia/Intervention 0=New York/Comparison |
| **10** | time | Num | 8 | Data Collection Timepoint 0=Baseline 1=Follow-Up |
